# Analysis of clinical and heart rate variability parameters associated with single-vessel and multivessel coronary artery disease: A retrospective study

**DOI:** 10.12669/pjms.42.9.20249

**Published:** 2026-09

**Authors:** Mingfang Zhu, Sixin Xie, Gang Lv, Xiang Zhang, Ding Peng

**Affiliations:** 1 Mingfang Zhu, Department of ECG Diagnosis, Ningguo People’s Hospital, Ningguo, Anhui Province 242300, P.R. China; 2 Sixin Xie, Department of Cardiac Function Examination, First Hospital of Quanzhou Affiliated to Fujian Medical University, Quanzhou, Fujian Province 362000, P.R. China; 3 Gang Lv, Department of Cardiology, Ningguo People’s Hospital, Ningguo, Anhui Province 242300, P.R. China; 4 Xiang Zhang, Department of Electrocardiographic Diagnosis, Xi’an Children’s Hospital, Xi’an, Shaanxi Province 710003, P.R. China; 5 Ding Peng Department of Cardiology, The Affiliated Qingyuan Hospital, Qingyuan People’s Hospital, Guangzhou Medical University, Qingyuan, Guangdong Province 511518, P.R. China

**Keywords:** Cardiac biomarkers, Coronary artery disease, Heart rate variability, Multivessel disease, SDNN

## Abstract

**Objectives:**

To compare heart rate variability (HRV) parameters and cardiac biomarkers between patients with single-vessel and multivessel coronary artery disease (CAD) and assess their associations with multivessel involvement.

**Methodology:**

This retrospective study included 196 patients with angiographically confirmed CAD admitted between September 2022 and May 2025 at Ningguo People’s Hospital. Patients were classified as having single-vessel (n=55) or multivessel (n=141) disease. Group differences were assessed, and logistic regression was used to examine associations of SDNN, deceleration capacity (DC), high-sensitivity cardiac troponin T (hs-TnT), and N-terminal pro-B-type natriuretic peptide (NT-proBNP) with multivessel disease. Restricted cubic spline analysis explored potential nonlinear relationships.

**Results:**

The multivessel group had lower SDNN, SDANN, SDNN index, and DC and higher hs-TnT and NT-proBNP levels than the single-vessel group (all P<0.05). After adjustment for age, sex, hypertension, and diabetes, higher SDNN was associated with lower odds of multivessel disease (OR=0.68, 95% CI: 0.49–0.94), whereas higher hs-TnT (OR=2.43, 95% CI: 1.35–4.37) and NT-proBNP (OR=3.51, 95% CI: 1.28–9.62) were associated with higher odds. DC was not significantly associated with multivessel disease. Spline analyses suggested possible nonlinear patterns for SDNN and DC, but these findings were exploratory.

**Conclusion:**

Lower SDNN and higher hs-TnT and NT-proBNP were associated with multivessel CAD. The hs-TnT finding should be interpreted in the context of myocardial injury burden and limited control of confounding.

## INTRODUCTION

Incidence of coronary artery disease (CAD), the leading cause of cardiovascular mortality worldwide, is on the rise due to the gradually aging population and changes in lifestyle.^1^ Clinically, CAD can be classified as single-vessel or multi-vessel disease based on the number of coronary arteries involved by lesions.^2^ Previous studies have reported that multi-vessel disease, which accounts for 58.3% of patients with CAD, is associated with a wider range of myocardial ischemia, more complex conditions, and a significantly higher prevalence of complications and risk factors compared to single-vessel disease.^3^,^4^ Nevertheless, the optimal timing and therapeutic strategies for myocardial revascularization in patients with multi-vessel disease remain unclear.^4^ Therefore, identifying factors associated with the extent of coronary artery involvement may provide clinically useful information for patient assessment and individualized treatment planning.

At present, traditional risk factors for CAD include age, gender, hypertension, diabetes, and dyslipidemia.^5^ However, among patients with angiographically confirmed coronary stenosis, these conventional risk factors may show substantial overlap, which limits their ability to distinguish single-vessel from multivessel disease.^6^ Heart rate variability (HRV) is a non-invasive indicator of cardiac autonomic nervous function; a marked reduction in HRV indicates sympathetic overactivation and parasympathetic inhibition, which are closely implicated in the occurrence and progression of CAD.^7^ Previous research has suggested that abnormal HRV parameters are associated with the extent and characteristics of coronary artery lesions.^8^ Nevertheless, the correlations of individual HRV indicators with the number of diseased coronary vessels and their dose-response relationships have not been fully clarified. Several studies have attempted to investigate the value of the deceleration capacity (DC), a vital index derived from HRV, in relation to the number of diseased coronary vessels,^9^,^10^ but conclusions remain inconsistent.

High-sensitivity cardiac troponin T (hs-TnT) is a specific biomarker of myocardial injury that enables the early identification of micro-myocardial ischemia, whereas N-terminal pro-B-type natriuretic peptide (NT-proBNP) primarily reflects the degree of cardiac dysfunction.^11^,^12^ Both biomarkers are widely applied in the evaluation of CAD, yet their associations with the number of diseased coronary vessels and the factors underlying these associations remain incompletely understood.

This study aimed to compare HRV parameters and laboratory indicators between patients with single-vessel and multivessel CAD, and to explore non-invasive markers associated with the extent of coronary artery involvement. These findings may provide additional information regarding the clinical characteristics associated with multivessel coronary involvement.

## METHODOLOGY

This retrospective observational study included clinical data from 196 patients with angiographically confirmed CAD who were admitted to Ningguo People’s Hospital between September 2022 and May 2025. The study population included patients with stable CAD and those presenting with acute coronary syndrome, including unstable angina and myocardial infarction, reflecting a real-world CAD cohort. All patients were definitively diagnosed with coronary artery disease via coronary angiography (CAG). According to the CAG findings, patients were divided into single-vessel (n=55) and multivessel (n=141) lesion groups. Single-vessel disease was defined as luminal stenosis ≥ 50% in only one of the four major coronary arteries, including the left main coronary artery, left anterior descending artery, left circumflex artery, and right coronary artery. Multi-vessel disease was defined as luminal stenosis ≥ 50% in two or more of the above coronary arteries. Under this vessel-count definition, isolated left main coronary artery stenosis was classified as single-vessel disease. Coronary angiograms were independently reviewed by two experienced cardiologists who were blinded to the HRV and laboratory results. Disagreements regarding vessel involvement or stenosis severity were resolved by consensus, with consultation from a senior cardiologist when necessary.

### Ethical approval:

The study was approved by the Ethics Committee of Ningguo People’s Hospital on April 27, 2026 (No. KS-035), and the requirement for informed consent was waived because of the retrospective nature of the study.

### Inclusion Criteria:

- Met the diagnostic criteria for CAD and confirmed by coronary angiography.
- Complete clinical data, HRV records, and laboratory examination results.
- Age ≥ 18 years old.

### Exclusion Criteria:

- Severe heart failure (New York Heart Association functional class IV), third-degree atrioventricular block, atrial fibrillation or atrial flutter, sustained ventricular tachycardia, pacemaker rhythm or an implanted cardiac pacemaker, or Holter recordings unsuitable for reliable HRV analysis.
- Severe valvular heart disease, malignant tumors, autoimmune diseases, or acute infectious diseases.

### Data collection:

Baseline clinical characteristics (age, gender, underlying chronic diseases, etc.), HRV parameters, and laboratory test indicators were extracted from the hospital electronic medical record system. Available information on cardiovascular medication use was extracted from the medical records; however, details regarding medication type, dose, duration, and recent dose changes were not consistently available.

### Detection of HRV parameters:

All patients underwent 24 hours ambulatory electrocardiography within one week after admission in a quiet, resting state using a 12-lead Holter recorder (DMS300-4A or DMS300-4AL, Dimu Software [Beijing] Co., Ltd.). The exact interval between admission and Holter monitoring was not consistently available in the retrospective records. During Holter monitoring, patients were instructed to avoid strenuous exercise and emotional agitation. Chronic cardiovascular medications, including beta-blockers when clinically indicated, were not routinely discontinued; however, no new initiation or acute dose adjustment of heart rate–affecting medications was documented during the 24 hours Holter monitoring period. Automatic analysis of Holter data was performed by a professional electrocardiographic analysis system. The extracted HRV indicators included time-domain parameters: standard deviation of all normal sinus RR intervals (SDNN), standard deviation of the average normal RR intervals every five minutes (SDANN), SDNN index, root mean square of successive RR interval differences (rMSSD), percentage of adjacent RR interval differences greater than 50 ms (pNN50), triangular index, and DC. All recordings were reviewed by trained electrocardiography personnel. HRV parameters were extracted and quality-controlled in accordance with established international standards for HRV measurement, physiological interpretation, and clinical use.^12^

### Laboratory biomarker detection:

Fasting peripheral venous blood (2–3 mL) was collected in the early morning on the next day after admission and placed into EDTA anticoagulant tubes. After centrifugation at 3000 r/min for ten minutes, plasma was collected for analysis. The level of hs-TnT was measured by an automatic chemiluminescence immunoassay analyzer (Cobas E601, Roche Diagnostics). NT-proBNP was detected using an automatic chemiluminescence analyzer (Autolumo A2000, Autobio Diagnostics).

### Statistical analysis:

Statistical analysis was performed using SPSS 27.0 and R 4.2.1 software. Non-normally distributed continuous variables were presented as medians and interquartile ranges, and between-group differences were compared using the Wilcoxon rank-sum test. Categorical variables were expressed as counts and percentages, and the chi-square test was used for intergroup comparisons. Logistic regression models were established to explore the associations between selected indicators and multivessel coronary lesions, including an unadjusted model, an age- and sex-adjusted model, and a multivariable model adjusted for age, sex, hypertension, and diabetes mellitus. Age, sex, hypertension, and diabetes mellitus were included as prespecified clinical covariates. Other potentially important confounders, including ACS status, prior myocardial infarction, renal function, LVEF, smoking, dyslipidemia, and medication use, could not be reliably incorporated because the relevant data were incomplete or unavailable. Restricted cubic spline regression, adjusted for age, sex, hypertension, and diabetes mellitus, was used as an exploratory analysis to assess potential dose–response patterns between continuous variables (SDNN, DC, hs-TnT, and NT-proBNP) and the probability of multivessel disease. Nonlinearity was assessed using the P value for nonlinearity. A two-tailed *P* value < 0.05 was considered statistically significant.

## RESULTS

A total of 196 patients with coronary artery disease were enrolled in the present study, including 55 cases of single-vessel disease and 141 cases of multi-vessel disease. The comparisons of baseline characteristics between the two groups are presented in Table-I. There were no significant differences in age, gender, hypertension, and diabetes distribution between the two groups (all P>0.05). Compared with the single-vessel disease group, SDNN, SDANN, SDNN index, and DC were significantly lower in the multivessel disease group (all P<0.05). Levels of hs-TnT and NT-proBNP were significantly higher in the multivessel disease group than in the single-vessel disease group (both P<0.001). Both groups had comparable rMSSD, pNN50, and triangular index (all P>0.05).

**Table-I:** Baseline characteristics of patients with single-vessel and multi-vessel coronary artery disease.

Variable	Single Vessel (N=55)	Multi Vessel (N=141)	Z/χ²	P-value
Age (years)	63.00 [55.00-71.00]	64.00 [56.00-71.00]	0.845	0.398
SDNN (ms)	111.00 [97.50-127.00]	94.00 [78.00-118.00]	2.958	0.003
SDANN (ms)	87.00 [75.50-105.00]	74.00 [60.00-95.00]	2.681	0.007
SDNN Index (ms)	60.00 [45.50-68.00]	51.00 [39.00-63.00]	2.415	0.016
rMSSD (ms)	35.00 [24.00-60.00]	32.00 [22.00-50.00]	1.557	0.119
pNN50 (%)	6.00 [2.00-14.50]	4.00 [2.00-10.00]	1.587	0.112
Triangular Index	27.00 [23.50-32.05]	24.10 [18.60-32.20]	1.928	0.054
DC (ms)	6.14 [5.25-7.66]	5.82 [4.18-6.58]	2.054	0.040
hs-TnT (ng/L)	23.68 [7.43-331.40]	446.50 [20.32-2003.00]	3.847	<0.001
NT-proBNP (pg/mL)	184.00 [112.68-490.31]	604.32 [201.10-1245.30]	3.845	<0.001
Gender (Male), n(%)	39 (70.9%)	106 (75.2%)	0.186	0.667
Diabetes (Yes), n(%)	8 (14.5%)	38 (27.0%)	2.734	0.098
Hypertension (Yes), n(%)	37 (67.3%)	88 (62.4%)	0.222	0.638

***Notes:*** Values are presented as median [interquartile range] or n (%), as appropriate. HRV, heart rate variability; SDNN, standard deviation of normal-to-normal intervals; SDANN, standard deviation of the averages of normal-to-normal intervals in all 5-min segments; SDNN index, mean of the standard deviations of all normal-to-normal intervals for all 5-min segments; rMSSD, root mean square of successive differences; pNN50, percentage of adjacent normal-to-normal intervals differing by more than 50 ms; DC, deceleration capacity; hs-TnT, high-sensitivity troponin T; NT-proBNP, N-terminal pro-B-type natriuretic peptide.

Logistic regression analysis was then done to evaluate the associations of SDNN, DC, hs-TnT, and NT-proBNP with multi-vessel disease. As shown in Table-II, in the unadjusted model, each 1-standard-deviation increase in SDNN was associated with 30% lower odds of multivessel disease (OR=0.70, 95% CI: 0.51–0.96, P=0.025), and each standard deviation increase in hs-TnT and NT-proBNP was associated with higher odds of multivessel disease (OR=2.19, 95% CI: 1.26–3.81, P=0.005; OR=3.57, 95% CI: 1.31–9.71, P=0.013). No significant association was observed between DC and multivessel disease (OR=0.82, 95% CI: 0.60–1.11, P=0.198). After adjustment for age and gender, the significant associations of SDNN, hs-TnT, and NT-proBNP with multi-vessel disease remained stable. In the multivariable model adjusted for age, sex, hypertension, and diabetes mellitus, SDNN remained negatively associated with multivessel disease (OR=0.68, 95% CI: 0.49–0.94, P=0.019), whereas hs-TnT (OR=2.43, 95% CI: 1.35–4.37, P=0.003) and NT-proBNP (OR=3.51, 95% CI: 1.28–9.62, P=0.015) were positively associated with multivessel disease. DC was not significantly associated with multivessel disease in any of the logistic regression models. Detailed regression parameters are provided in Supplementary Table-SI.

**Table-II:** Associations of SDNN, DC, hs-TnT, and NT-proBNP with multi-vessel coronary artery disease in logistic regression models.

Exposure Variable	Model I OR (95% CI)	Model I P-value	Model II OR (95% CI)	Model II P-value	Model III OR (95% CI)	Model III P-value
SDNN (per 1-SD)	0.70 (0.51-0.96)	0.025	0.69 (0.50-0.95)	0.021	0.68 (0.49-0.94)	0.019
DC (per 1-SD)	0.82 (0.60-1.11)	0.198	0.82 (0.60-1.11)	0.199	0.80 (0.59-1.10)	0.166
hs-TnT (per 1-SD)	2.19 (1.26-3.81)	0.005	2.32 (1.30-4.13)	0.004	2.43 (1.35-4.37)	0.003
NT-proBNP (per 1-SD)	3.57 (1.31-9.71)	0.013	3.30 (1.22-8.93)	0.019	3.51 (1.28-9.62)	0.015

***Notes:*** Model 1 was unadjusted. Model 2 was adjusted for age and sex. Model 3 was adjusted for age, sex, hypertension, and diabetes mellitus. Odds ratios were estimated per 1-standard deviation increase in each variable. OR, odds ratio; CI, confidence interval; SDNN, standard deviation of normal-to-normal intervals; DC, deceleration capacity; hs-TnT, high-sensitivity troponin T; NT-proBNP, N-terminal pro-B-type natriuretic peptide.

**Supplementary Table-SI:** Detailed regression parameters for logistic models assessing the associations of SDNN, DC, hs-TnT, and NT-proBNP with multi-vessel coronary artery disease.

Exposure Variable	Model	β (Coefficient)	SE	Z-value	OR (95% CI)	P-value
SDNN (per 1-SD)	Model I (Crude)	-0.3602	0.1604	-2.2450	0.70 (0.51-0.96)	0.025
SDNN (per 1-SD)	Model II (Adjusted)	-0.3697	0.1604	-2.3048	0.69 (0.50-0.95)	0.021
SDNN (per 1-SD)	Model III (Multivariable Adjusted)	-0.3851	0.1636	-2.3536	0.68 (0.49-0.94)	0.019
DC (per 1-SD)	Model I (Crude)	-0.1993	0.1548	-1.2872	0.82 (0.60-1.11)	0.198
DC (per 1-SD)	Model II (Adjusted)	-0.1992	0.1552	-1.2832	0.82 (0.60-1.11)	0.199
DC (per 1-SD)	Model III (Multivariable Adjusted)	-0.2209	0.1596	-1.3847	0.80 (0.59-1.10)	0.166
hs-TnT (per 1-SD)	Model I (Crude)	0.7835	0.2822	2.7769	2.19 (1.26-3.81)	0.005
hs-TnT (per 1-SD)	Model II (Adjusted)	0.8396	0.2954	2.8418	2.32 (1.30-4.13)	0.004
hs-TnT (per 1-SD)	Model III (Multivariable Adjusted)	0.8877	0.2999	2.9603	2.43 (1.35-4.37)	0.003
NT-proBNP (per 1-SD)	Model I (Crude)	1.2728	0.5103	2.4940	3.57 (1.31-9.71)	0.013
NT-proBNP (per 1-SD)	Model II (Adjusted)	1.1943	0.5077	2.3524	3.30 (1.22-8.93)	0.019
NT-proBNP (per 1-SD)	Model III (Multivariable Adjusted)	1.2559	0.5144	2.4417	3.51 (1.28-9.62)	0.015

***Notes:*** The table presents regression coefficients, standard errors, Wald statistics, odds ratios, 95% confidence intervals, and P values for each logistic regression model.

To further explore potential dose–response patterns between continuous variables and the probability of multivessel disease, exploratory restricted cubic spline models were used for SDNN, DC, hs-TnT, and NT-proBNP (Fig.1). The RCS curves suggested potential nonlinear patterns between SDNN and the probability of multivessel disease (P for nonlinearity =0.032, Fig.1A) and between DC and multivessel disease (P for nonlinearity =0.013, Fig.1B). In contrast, no significant nonlinear relationships were detected for hs-TnT and NT-proBNP (P for nonlinearity: 0.323 and 0.136, respectively; Fig.1C and 1D), and their overall trends indicated that elevated levels of both biomarkers were associated with a gradual increase in the estimated probability of multivessel disease. The SDNN and DC curves showed fluctuations in some value ranges, and these exploratory patterns should be interpreted cautiously. Although hs-TnT and NT-proBNP did not reach statistical significance for nonlinearity, their overall trends were consistent with a higher likelihood of multi-vessel disease as their concentrations increased.

**Fig.1:**
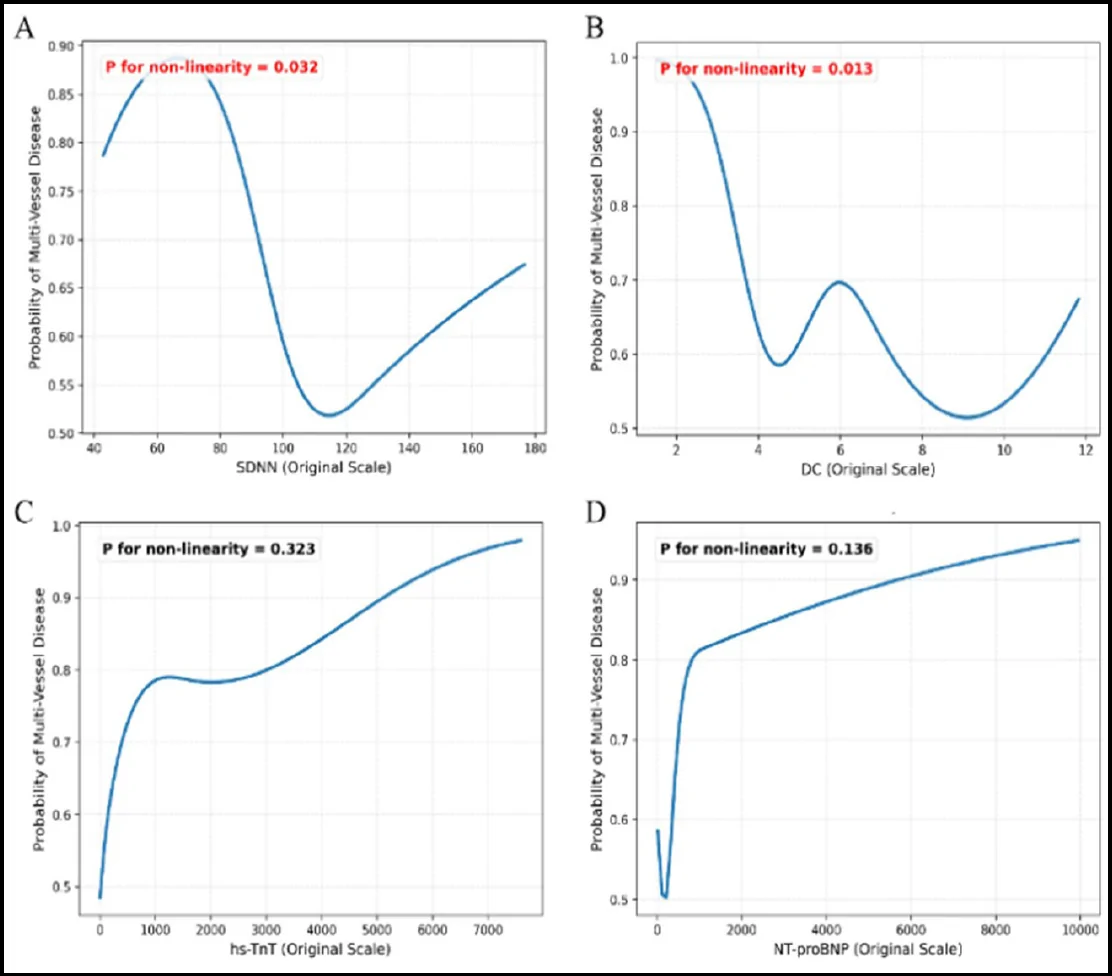
Restricted cubic spline analyses of the associations between SDNN, DC, hs-TnT, NT-proBNP, and the probability of multi-vessel coronary artery disease. (A) SDNN; (B) DC; (C) hs-TnT; (D) NT-proBNP. The curves depict the exploratory adjusted associations between each continuous variable and the probability of multivessel coronary artery disease. The RCS curves are presented as exploratory analyses only. Because confidence intervals and data-density distributions are not displayed, the apparent curve shapes, particularly for DC, should be interpreted cautiously and should not be considered evidence of a stable nonlinear association.

## DISCUSSION

This study showed that SDNN was lower, whereas hs-TnT and NT-proBNP were higher, in patients with multivessel CAD. After adjustment for age, sex, hypertension, and diabetes mellitus, higher SDNN was associated with lower odds of multivessel disease, whereas higher hs-TnT and NT-proBNP were associated with higher odds. Exploratory RCS analyses suggested potential nonlinear patterns for SDNN and DC, although the DC finding was not supported by the logistic regression models. These findings relate specifically to the number of diseased vessels and should be interpreted in light of the limited covariate adjustment and the heterogeneous clinical presentations included in the cohort.

This study demonstrated that SDNN was significantly reduced in the multivessel disease group and remained negatively associated with multivessel disease in multivariable analysis, suggesting that weakened overall autonomic nervous regulation may be related to broader coronary artery involvement. These results are consistent with previous studies. Van Boven et al.^13^ reported that lower SDNN was related to subsequent adverse events in patients with stable CAD. Feng et al.^14^ found that SDNN and SDANN declined gradually with increasing numbers of diseased coronary vessels, with the decline most pronounced in patients with three-vessel disease. Although Liu et al.^15^ failed to identify a significant direct correlation between HRV and lesion number, they observed a negative correlation between SDANN and the Gensini score, implying that HRV may better reflect global atherosclerotic burden rather than simply the number of stenotic vessels. Such discrepancies in reported outcomes may be attributed to differences in study populations, diabetic status, previous myocardial injury, and selection of HRV indicators.^13^-^15^

Decreased SDNN is generally considered to reflect impaired autonomic modulation, including relatively enhanced sympathetic activity and attenuated vagal tone, although HRV parameters may also be influenced by clinical status and background medications.^16^ Autonomic imbalance may contribute to coronary vasomotor dysregulation, disturbance of the myocardial oxygen supply–demand balance, and progression of atherosclerosis.^17^ A study by Watanabe et al.^18^ confirmed the link between HRV and endothelial function in patients with ischemic heart disease, and a study by Sunbul et al.^19^ showed that reduced HRV was frequently associated with impaired endothelial function in patients with CAD. Studies have also highlighted the critical role of the neuroimmune axis in inflammatory amplification, plaque stability, and vascular wall immune regulation.^20^,^21^ These mechanisms may provide a plausible biological context for the observed association between lower SDNN and multivessel disease.^17^–^21^

In the present study, DC was lower in the multivessel disease group, but it was not significantly associated with multivessel disease in any of the logistic regression models. Although the exploratory RCS analysis suggested a possible nonlinear pattern, this finding does not establish a stable or independent association. DC primarily reflects cardiac deceleration capacity and vagal modulation and may be influenced by cardiac function, metabolic status, inflammation, and background treatment.^9^,^22^–^24^ These factors may partly explain the variability in DC measurements, but the present data are insufficient to establish a biological mechanism underlying the observed spline shape. The nonlinear pattern should therefore be regarded as exploratory and requires validation in larger independent cohorts.

This study showed that hs-TnT was positively associated with multivessel CAD after adjustment for the available clinical covariates. This finding suggests that higher hs-TnT reflects a greater myocardial injury burden in the multivessel group, but it should not be interpreted as a direct anatomical marker of the number of diseased coronary vessels. These results are highly consistent with the findings reported by Ndrepepa et al.^25^ In a stable CAD cohort, hs-TnT increased stepwise in patients with normal coronary arteries, single-vessel, two-vessel, and three-vessel disease, and this correlation remained significant after adjustment for traditional risk factors, NT-proBNP, and C-reactive protein (CRP). These observations suggest that even among patients with confirmed CAD, hs-TnT may provide information related to the extent of coronary involvement and myocardial injury burden.

Higher hs-TnT levels may arise from several overlapping processes, including acute ischemic injury, myocardial infarction, recurrent ischemia, and a greater burden of jeopardized myocardium. Therefore, the marked between-group difference in hs-TnT cannot be attributed solely to the number of diseased coronary vessels.^25^ Because the cohort included both stable CAD and acute coronary presentations, the markedly higher hs-TnT level in the multivessel group should also be interpreted in the context of possible differences in acute myocardial injury burden. Therefore, hs-TnT should be regarded as a marker of myocardial injury burden rather than a purely anatomical indicator of vessel count.^25^

NT-proBNP levels in this study were also positively associated with multivessel CAD after adjustment for available covariates, indicating that multivessel coronary involvement may be accompanied by increased myocardial wall stress and a greater global cardiovascular burden. In a previous prognostic modeling study, Holtrop et al.^26^ identified NT-proBNP as an additional predictor in the SMART2 model, while multivessel coronary disease also contributed prognostic information. Although NT-proBNP is widely used in the diagnosis and assessment of heart failure, it also reflects myocardial wall stress and provides diagnostic and prognostic information across a range of cardiovascular conditions.^27^ Köster et al.^28^ demonstrated a close relationship between NTproBNP and coronary artery disease burden. Elevated NT-proBNP in patients with multivessel disease may reflect greater myocardial wall stress and overall cardiac burden.^26^–^28^

The exploratory RCS analyses suggested potential nonlinear patterns between SDNN and DC and the probability of multivessel disease, whereas hs-TnT and NT-proBNP showed approximately monotonic increasing trends without significant evidence of nonlinearity. The finding for DC should be interpreted with particular caution because DC was not significantly associated with multivessel disease in any of the logistic regression models. This discrepancy suggests that the apparent nonlinear pattern may be data-dependent and should not be interpreted as evidence of an independent association. In addition, fluctuations in the SDNN and DC curves may partly reflect sparse data distribution in certain value ranges and the limited sample size, rather than a definitive biological threshold effect.

### Strengths of this study:

The strengths of this study include comprehensive intergroup comparison, multivariate regression analysis, and further exploration of dose–response relationships via RCS models. The study evaluated HRV parameters, hs-TnT, and NT-proBNP within the same cohort, allowing their individual associations with multivessel disease to be considered from the perspectives of autonomic regulation, myocardial injury, and cardiac stress. Additionally, all enrolled patients received a definite diagnosis by coronary angiography, which strengthens the clinical pertinence and applicability of our findings.

### Limitations:

First, it was a single-center retrospective study with a relatively small sample size, which limits causal inference and the generalizability of the findings. The restricted cubic spline analyses were exploratory, and the observed curve shapes may have been influenced by the limited sample size and uneven data distribution. This concern is particularly relevant to DC, which was not significantly associated with multivessel disease in any logistic regression model; therefore, its apparent nonlinear pattern should not be interpreted as evidence of an independent association or a clinically meaningful threshold. Second, residual confounding could not be excluded. Although age, sex, hypertension, and diabetes mellitus were included in the multivariable models, several clinically relevant factors, including ACS status, prior myocardial infarction, renal function, LVEF, smoking, dyslipidemia, and cardiovascular medication use, were incomplete or unavailable. In particular, the use of beta-blockers, antiarrhythmic drugs, and other heart rate–modifying medications could not be fully characterized or adjusted for and may have influenced the HRV findings. The timing of Holter monitoring within the first week after admission was also not standardized, and changes in clinical status or treatment before monitoring may have affected HRV parameters. Third, the cohort included both stable CAD and acute coronary presentations, but the distributions of stable CAD, unstable angina, and myocardial infarction could not be reliably reconstructed or compared between groups. Consequently, stratified or adjusted analyses according to clinical presentation were not feasible. This limitation is especially relevant to hs-TnT and NT-proBNP, because differences in acute myocardial injury and cardiac stress may have contributed to the observed between-group differences and may have led to overestimation of their associations with vessel count. Fourth, coronary involvement was classified solely according to the number of diseased vessels. This approach did not fully capture lesion location, stenosis severity, diffuse disease, or overall anatomical complexity. Gensini and SYNTAX scores were unavailable and could not be reliably reconstructed. In addition, the vessel-count classification did not fully reflect the distinct anatomical and prognostic significance of left main coronary artery disease. Because isolated left main disease was uncommon, a separate sensitivity analysis was not performed. Therefore, the present findings should be interpreted as associations with vessel count rather than with the overall severity or complexity of coronary atherosclerosis. Finally, long-term follow-up data were unavailable, and no combined model incorporating HRV parameters, hs-TnT, and NT-proBNP was developed or validated. Accordingly, this study could not assess subsequent cardiovascular outcomes or determine the discriminatory, calibration, or incremental value of combining these indicators.

## CONCLUSION

In patients with angiographically confirmed CAD, higher SDNN was associated with lower odds of multivessel disease, whereas higher hs-TnT and NT-proBNP were associated with higher odds after adjustment for the available clinical covariates. These findings relate to vessel count and should be interpreted cautiously given the potential influence of clinical presentation and residual confounding. Further studies are needed to evaluate the independent and combined clinical value of these indicators.

### Recommendations:

Future multicenter prospective studies with larger sample sizes should incorporate more complete clinical, functional, renal, medication, and coronary anatomical data, including validated scores such as the Gensini or SYNTAX score, to confirm the stability and clinical relevance of these associations.
